# Attitudes towards AI: measurement and associations with personality

**DOI:** 10.1038/s41598-024-53335-2

**Published:** 2024-02-05

**Authors:** Jan-Philipp Stein, Tanja Messingschlager, Timo Gnambs, Fabian Hutmacher, Markus Appel

**Affiliations:** 1 Department of Media Psychology, Institute for Media Research, Chemnitz University of Technology, Thüringer Weg 11, 09126 Chemnitz, Germany; 2 Psychology of Communication and New Media, Human-Computer-Media Institute, University of Würzburg, Würzburg, Germany; 3 Leibniz Institute for Educational Trajectories, Bamberg, Germany

**Keywords:** Psychology, Human behaviour, Information technology

## Abstract

Artificial intelligence (AI) has become an integral part of many contemporary technologies, such as social media platforms, smart devices, and global logistics systems. At the same time, research on the public acceptance of AI shows that many people feel quite apprehensive about the potential of such technologies—an observation that has been connected to both demographic and sociocultural user variables (e.g., age, previous media exposure). Yet, due to divergent and often ad-hoc measurements of AI-related attitudes, the current body of evidence remains inconclusive. Likewise, it is still unclear if attitudes towards AI are also affected by users’ personality traits. In response to these research gaps, we offer a two-fold contribution. First, we present a novel, psychologically informed questionnaire (ATTARI-12) that captures attitudes towards AI as a single construct, independent of specific contexts or applications. Having observed good reliability and validity for our new measure across two studies (*N*_1_ = 490; *N*_2_ = 150), we examine several personality traits—the Big Five, the Dark Triad, and conspiracy mentality—as potential predictors of AI-related attitudes in a third study (*N*_3_ = 298). We find that agreeableness and younger age predict a more positive view towards artificially intelligent technology, whereas the susceptibility to conspiracy beliefs connects to a more negative attitude. Our findings are discussed considering potential limitations and future directions for research and practice.

## Introduction

Artificial intelligence (AI) promises to make human life much more comfortable: It may foster intercultural collaboration via sophisticated translating tools, guide online customers towards the products they are most likely going to buy, or carry out jobs that feel tedious to human workers^1,2^. At the same time, the proliferation of AI-based technologies is met with serious reservations. It is argued, for instance, that AI could lead to the downsizing of human jobs^3,4^, the creation of new intelligent weaponry^5,6^, or a growing lack of control over the emerging technologies^7^. Similarly, the recent and much-publicized presentation of new text- and image-creating AI (such as *ChatGPT* and *Midjourney*) has raised concerns about artistic license, academic fraud, and the value of human creativity^8^.

Crucially, individuals may differ regarding their evaluation of such chances and risks, and in turn, hold different attitudes towards AI. However, we note that scholars interested in these differences often used a basic ad-hoc approach to assess participants’ AI attitudes^6,9^, or focused only on highly domain-specific sub-types of AI^10,11^. Against this background, the current paper offers a twofold contribution. First, we present a novel questionnaire on AI-related attitudes—the ATTARI-12, which incorporates the psychological trichotomy of cognition, emotion, and behavior to facilitate a comprehensive yet economic measurement—and scrutinize its validity across two studies. Subsequently, we connect participants’ attitudes (as measured by our new scale) to several fundamental personality traits, namely the Big Five, the Dark Triad, and conspiracy mentality.

## Research objective 1: measuring attitudes towards AI

Broadly speaking, the term *artificial intelligence* (AI) is used to describe technologies that can execute tasks one may expect to require human intelligence—i.e., technologies that possess a certain degree of autonomy, the capacity for learning and adapting, and the ability to handle large amounts of data^12,13^. As AI becomes deeply embedded in many technical systems and, thus, people’s daily lives, it emerges as an important task for numerous scientific disciplines (e.g. psychology, communication science, computer science, philosophy) to better understand users’ response to—and acceptance of—artificially intelligent technology. Of course, in order to achieve this goal, it is essential to employ theoretically sound measures of high psychometric quality when assessing AI-related attitudes. Furthermore, researchers need to decide whether they want to examine only a specific type of application (such as self-driving cars or intelligent robots) or focus on AI as an abstract technological concept that can be applied to many different settings. While both approaches have undeniable merit, it may be argued that the comparability of different research efforts—especially those situated at the intersection of different disciplines—clearly benefits from the latter, i.e., an empirical focus on attitudes towards AI as a set of technological capabilities instead of concrete use cases.

In line with this argument, scientific efforts have produced several measures to assess attitudes towards AI as a more overarching concept. These include the General Attitudes Towards Artificial Intelligence Scale (GAAIS^13^), the Attitudes Towards Artificial Intelligence Scale (ATAI^14^), the AI Anxiety Scale (AIAS^15^), the Threats of Artificial Intelligence Scale (TAI^16^), and the Concerns About Autonomous Technology questionnaire^17^. Upon thorough examination, however, we believe that none of these measures offer an entirely satisfactory option for at least one of five reasons. First, many of the above-mentioned measures only inquire participants about negative impressions and concerns regarding AI, leaving aside the possibility of distinctly positive attitudes^15–17^. Second, the measured attitudes towards AI are often subdivided into several factors^13–15^, which complicates handling and interpreting these scales from both a theoretical and a practical perspective. For instance, the GAAIS^13^ presents two sub-scales called “acceptance” and “fear,” even though it can be argued that these merely cover different poles of the same spectrum. Relatedly, the AIAS scale^15^ offers a four-factor solution with the (statistically induced) dimensions “learning,” “job replacement,” “sociotechnical blindness,” and “AI configuration”—clusters whose practical merit may be related to more specific use cases. Third, while some scales have a relatively low number of items (e.g., five items^14^), other scales are comparably long (e.g., 21 items^15^), which may complicate their use in research settings that need to include attitudes towards AI as one concept among multiple others. Fourth, for one of the reviewed scales^14^, the low number of items resulted in a notable lack of reliability. Fifth, none of the available scales acknowledge the cognitive, affective, and behavioral facets of attitudes in their designs^18,19^.

Hence, based on the identified lack of a one-dimensional, psychometrically sound, yet economic measure that captures both positive and negative aspects of the attitude towards AI, our first aim was to develop a scale that overcomes these limitations. For the sake of broad interdisciplinary applicability, we explicitly focused the creation of our scale on the perception of AI as a general set of technological capabilities, independent of its physical embodiment or use context. This perspective was not least informed by recent research, which indicated that people’s evaluation of digital ‘minds’ is likely to change once visual factors come into play^20^—further highlighting the merit of addressing AI as a disembodied concept.

## Research objective 2: understanding associations between AI-related attitudes and personality traits

Thus far, most studies on public AI acceptance have explored the role of demographic and sociocultural variables, revealing that concerns about AI seem to be more prevalent among women, the elderly, ethnic minorities, and individuals with lower levels of education^6,21^. Furthermore, it was demonstrated that egalitarian values and distrust in science^9^, attachment anxiety^22^, and the exposure to dystopian science fiction^6,23^ constitute meaningful predictors of negative attitudes towards AI. Indeed, many of these findings are echoed by a large body of human–robot interaction literature, which also emphasizes the crucial impact of demographic variables, cultural norms, and media use on the public acceptance of robotic machinery^24–26^. Yet, considering that studies from this discipline rarely elucidate whether the reported effects arise from the perception of robotic bodies or robotic minds (i.e., AI), we suggest that they should only be considered as secondary evidence for the current topic.

Apart from considerable insight into the sociocultural predictors of (dis-)liking AI technology, much less is known about the impact of users’ personality factors on their AI-related attitudes. To our knowledge, only a handful of scientific studies have actually addressed this issue, and most of them have yielded rather limited results—either by focusing on very specific types of AI^10,11,27^ or by measuring reactions to physically embodied AI (e.g., robots), which makes it much harder to pinpoint the cause of the effect^28,29^. While another recent study indeed explored attitudes towards AI as a technical concept regardless of its specific use or embodiment^30^, this effort focused exclusively on negative attitudes, thus leaving out a substantial part (i.e., the positive side) of how users think and feel about artificially intelligent systems.

Therefore, building upon the reviewed work, our second objective was to scrutinize several central personality dimensions as predictors for people’s AI-related attitudes, including the well-known Big Five^31^ and Dark Triad of personality^32^. In addition to that, we include the rather novel construct of conspiracy mentality in our work^33^, considering its high contemporary relevance in an increasingly digital world^34^.

## Overview of studies and predictions

Addressing the outlined research propositions, we present three studies (see Table 1 for a brief overview of main study characteristics). All materials, obtained data, and analysis codes for these three studies can be found in our project’s Open Science Framework repository (https://osf.io/3j67a/). Informed consent was collected from all participants before they took part in our research efforts.

**Table 1:** Overview of the conducted studies.

	Study 1	Study 2	Study 3
Sample type	MTurk (US)	Student sample	MTurk (US)
Study focus	Scale development: Factorial validity, reliability (internal consistency), construct validity	Scale development: Reliability (re-test reliability), construct validity	Personality predictors (Big Five, Dark Triad, conspiracy mentality)
Study language	English	German	English
Final sample size	490	166 (at Time 1), 163 at Time 2, 150 at both times	298
Measurements	1	2 (four to five week follow-up)	1

Studies 1 and 2 mainly served to investigate the reliability and validity of our new attitudes towards AI scale, the ATTARI-12. Hence, these studies were mainly guided by several analytical steps examining the statistical properties of our measure, including its convergent validity, re-test reliability, and susceptibility to social desirability bias. In Study 3, we then set out to connect participants’ attitude towards AI (as measured by the developed questionnaire) to fundamental personality traits and demographic factors. For this third and final study, we will detail all theoretical considerations and hypotheses in the following.

Faced with a lack of previous findings regarding personality predictors of AI-related attitudes, we deemed it important to anchor our investigation in a broader view at human personality. This perspective led us to first include the Big Five model^31^, which has remained the most widely used taxonomy of human personality for several decades. As the name suggests, the Big Five consist of five fundamental personality dimensions—openness to experience, conscientiousness, extraversion, agreeableness, and neuroticism—which are presumed to cover a substantial portion of the dispositional variance between individuals^35,36^. Adhering to this notion, we developed a first set of hypotheses about the individual contribution of each Big Five trait to people’s AI-related attitudes.

For *openness to experience*—which can be defined as the tendency to be adventurous, intellectually curious, and imaginative—we expected a clear positive relationship to AI-related attitudes. Since AI technologies promise many new possibilities for human society, people who are open to experience should feel more excited and curious about the respective innovations (this is mirrored by recent findings on self-driving cars^11^). We hypothesize:

### H1

The higher a person’s openness for experience, the more positive attitudes they hold towards AI.

*Conscientiousness* can be described as the inclination to be diligent, efficient, and careful, and to act in a disciplined or even perfectionist manner. Considering that it may be difficult for humans to understand the inner workings of an AI system or to anticipate its behavior, it seems likely that conscientious people would express more negative views about AI, a technological concept that might make the world less comprehensible for them^29^.

### H2

The higher a person’s conscientiousness, the more negative attitudes they hold towards AI.

The third of the Big Five traits, *extraversion*, encompasses the tendency to be out-going, talkative, and gregarious; in turn, it is negatively related to apprehensive and restrained behavior. Based on this definition, it comes as no surprise that extroverted individuals reported fewer concerns about autonomous technologies in previous studies^29,30^,^37^. Thus, we hypothesize:

### H3

The higher people’s extraversion, the more positive attitudes they hold towards AI.

High *agreeableness* manifests itself in the proclivity to show warm, cooperative, and kind-hearted behavior. Regarding the topic at hand, previous research suggests that agreeableness might be (moderately) correlated with positive views about automation or specific types of AI^11,27,30^. In line with this, we assume:

### H4

The higher a person’s agreeableness, the more positive attitudes they hold towards AI.

The last Big Five trait, *neuroticism*, is connected to self-conscious and shy behavior. People with high scores in this trait tend to be more vulnerable to external stressors and have trouble controlling spontaneous impulses. MacDorman and Entezari^28^ discovered that neurotic individuals reported significantly stronger eeriness after they had encountered an autonomous android than those with lower scores in the trait. Similar findings emerged with regards to people’s views on self-driving cars^11^ and narrow AI applications^30^. Based on this evidence as well as the generally anxious nature of neurotic individuals, our hypothesis is as follows:

### H5

The higher a person’s neuroticism, the more negative attitudes they hold towards AI.

Although the Big Five are considered a relatively comprehensive set of human personality traits, research has yielded several other concepts that serve to make sense of interpersonal differences (and how they relate to attitudes and behaviors). Importantly, tapping into more negatively connotated aspects of human nature, Paulhus and Williams^32^ introduced the Dark Triad of personality, a taxonomy that gathers three malevolent character traits: Machiavellianism, psychopathy, and narcissism. Due to their antisocial qualities, the Dark Triad have been frequently connected to deviant behaviors, interpersonal problems, and increased difficulties in the workplace^38,39^. Moreover, they have become an important part of exploring interpersonal differences in attitude formation, not least including views on modern-day technology^10^. In consequence, our second set of hypotheses revolves around the potential influence of these rather vindictive personality traits.

*Machiavellianism* is a personality dimension that encompasses manipulative, callous, and amoral qualities. For our research topic, we contemplated two opposing notions as to how this trait could relate to AI attitudes. On the one hand, recent literature suggests that Machiavellian individuals might feel less concerned about amoral uses of AI and instead focus on its utilitarian value, which may reflect in more positive attitudes^10^. On the other hand, the prospect of AI-driven surveillance remains a prominent topic in the public discourse^40^—and we deemed it likely that people high in Machiavellianism would be wary of this, wanting for their more deviant actions to remain undetected. Weighing both arguments, we ultimately settled for the latter and hypothesized:

### H6

The higher a person’s Machiavellianism, the more negative attitudes they hold towards AI.

A person scoring high on *psychopathy* is likely prone to thrill-seeking behavior and may experience only little anxiety. Unlike the deliberate manipulations typical for Machiavellian people, the psychopathic personality trait involves much more impulsive antisocial tendencies. Based on this, we came to assume a positive relationship between psychopathy and attitudes towards AI—not least considering that our dependent variable would also involve people’s emotional reactions towards AI, which should turn out less fearful among those high in psychopathy. We assumed:

### H7

The higher a person’s psychopathy, the more positive attitudes they hold towards AI.

Broadly speaking, *narcissists* tend to show egocentric, proud, and unempathetic behavior, which is often accompanied by feelings of grandeur and entitlement. Whereas some qualitative research suggests that intelligent computers might deal a severe “blow to our narcissism” (^41^, p. 145), certain possibilities offered by AI could also appear quite attractive to people who score high in this trait—such as the futuristic notion of inserting aspects of oneself into a computer algorithm, to be preserved for all eternity. Surprisingly enough, inquiring participants about this very idea did not reveal a significant relation to narcissism in a recent study^10^. Then again, it should be noted that highly narcissistic individuals also tend to show open and extroverted behavior^32^, which would suggest a more positive relationship with AI-related attitudes depending on our other hypotheses. In summary, we settled on the following, cautiously positive assumption:

### H8

The higher a person’s narcissism, the more positive attitudes they hold towards AI.

Lastly, we proceeded to the exploration of a dispositional variable on a notably smaller scale of abstraction, which appeared promising to us from a contemporary perspective: people’s *conspiracy mentality*. Strongly related to general distrust in people, institutions, and whole political systems, this personality trait focuses on the susceptibility to *conspiracy theories*, i.e., explanations about famous events that defy common sense or publicly presented facts^33^. Psychological research has shown that people who believe in one conspiracy theory are typically more likely to also believe in another, indicating a stable trait-like tendency to subscribe to an overly skeptical and distrusting way of thinking^42^. Further emphasizing this angle, recent research has suggested that the tendency to accept epistemically suspect information might constitute a single dimension that further connects to difficulties in analytic thinking, an overestimation of one’s own knowledge, as well as receptivity for ‘pseudo-profound bullshit’^43^.

Although they might not be quite as well-known as other prominent conspiracy theories (e.g., concerning the 9/11 attacks), there are in fact several conspiracist beliefs about the use of sophisticated technology and AI. The respective theories range from the idea that those in power want to replace certain individuals with intelligent machines^44^ to the belief that COVID-19 vaccinations are actually injections of sophisticated nano-chips to establish a global surveillance system^45^. While these notions might seem somewhat obscure to many, the triumph of social media as a news source has presented proponents of conspiracy theories with a powerful platform to disseminate their ideas^46^. In addition to that, the well-documented (mis-)uses of big data—e.g., in the Cambridge Analytica scandal—has led many people to at least think about how computer algorithms might be used for sinister purposes^47^. Taking into account all of these observations, we anticipated that conspiracy mentality relates to more negative attitudes towards AI:

### H9

The higher a person’s level of conspiracy mentality, the more negative attitudes they hold towards AI.

Concluding the theoretical preparation of our study, we devised hypotheses on two basic demographic variables that were previously connected to AI-related attitudes in scientific publications. Specifically, it has been found that women typically hold more negative attitudes towards AI than men, which may, among other causes, be explained by societal barriers that limit women’s access to (and interest in) technical domains^6,21^. Likewise, studies have hinted at the higher apprehensiveness towards AI-powered technology among the elderly, potentially due to a lack of understanding and accessibility^6^. In the expectation to replicate these prior findings, we proposed:

### H10

Women hold more negative attitudes towards AI than men.

### H11

Older individuals hold more negative attitudes towards AI than younger individuals.

## Study 1

The goal underlying Study 1 was to develop a psychometrically sound scale that was (a) one-dimensional, (b) incorporated items reflecting the three bases or facets of attitudes (cognitive, affective, behavioral) that have guided attitude research in psychology for the last decades^18,19^, and (c) consisted of positively and negatively worded items to ensure that attitudes were measured on a full spectrum between aversion and enthusiasm (and agreement bias did not confound the results systematically). Based on theory and existing attitude scales in applied fields^13,48^, 24 original items were generated. Item generation was guided by the goals of developing an equal number of negatively and positively worded items and an equal number of items representing the three attitude facets (cognitive, affective, behavioral). Adhering to prior AI theory^49^ and existing measurement approaches^13^, the items were preceded by an instruction that introduced the phenomenon of artificial intelligence to participants, in order to reduce semantic ambiguities about the attitude target. This instruction is an integral part of the measurement.

In a second step, the authors discussed the items, and excluded items that were ambiguous in content, too specific in terms of attitude target, or involved too much linguistic overlap with other items. The remaining scale consisted of twelve items, with each of the three psychological facets of human attitudes—cognitive, affective, and behavioral—being represented by two positively and two negatively worded items. Nevertheless, all twelve items were expected to represent one general factor: People’s attitude towards AI. The full scale (ATTARI-12) can be found in the Appendix.

To gain insight into the validity of our new scale, Study 1 further included items on more specific AI applications. We expected that general attitudes towards AI as measured by the ATTARI-12 would be positively associated with specific attitudes towards electronic personal assistants (e.g., *Alexa*) and attitudes towards robots. Moreover, we assessed participants’ tendency to give socially desirable answers to establish whether our novel instrument would be affected by social desirability bias. Based on our careful wording during the construction of the scale, we expected that people’s attitude towards AI (per the ATTARI-12) would not be substantially related to social desirability. The hypotheses and planned data analysis steps for Study 1 were preregistered at https://aspredicted.org/8B5_GHZ (see also Supplement S1).

### Method

#### Ethics statement

In Germany, institutional ethics approval is not required for psychological research as long as it does not involve issues regulated by law^50^. All reported studies (Study 1, Study 2, and Study 3) were conducted in full accordance with the Declaration of Helsinki, as well as the ethical guidelines provided by the German Psychological Society (DGPs^51^). Of course, this also included obtaining informed consent from all participants before they were able to take part in this study.

#### Participants and procedure

Power analyses with *semPower* (Version 2.0.1^52^) suggested a necessary sample size of at least 500 respondents for the planned study. Therefore, at least 600 participants were aspired in order to have a proper data basis for our analyses—while still retaining a buffer for potential exclusions. As such, a total of 601 participants were recruited from the US-American *MTurk* participant pool. To ensure satisfactory data quality, we set our MTurk request to at least 500 previously completed tasks (also known as HITs), as well as > 98% HIT approval rate. Each participant was rewarded $1.00 for their participation, which lasted around three to five minutes.

Following preregistered exclusion criteria (i.e., completion time, failing at least one of two attention checks), 111 participants were excluded from our analyses. More specifically, 31 participants completed the questionnaire in less than 120 s, 79 participants did not describe the study as instructed, and one participant reported a year of birth that differed from the reported age by more than three years. Thus, the final sample consisted of 490 participants (212 female, 273 male, 5 other or no answer). The participants were between 19 and 72 years old (*M* = 39.78 years, *SD* = 11.06). For additional demographic information, please consult Supplement S2. After participants had given their informed consent, they completed a first attention check before proceeding to the ATTARI-12 questionnaire. Following this, we assessed their attitudes towards electronic personal assistants and robots, before presenting a measure of socially desirable answering. Lastly, participants had to summarize the study’s topic as an additional attention check and respond to several sociodemographic questions.

#### Measures

##### Attitudes towards artificial intelligence

We applied the newly created ATTARI-12, using a five-point answer format to capture participants’ responses (1 = *strongly disagree*, 5 = *strongly agree*). Descriptive measures and the reliability of the scale are reported in the Results section.

##### Attitudes towards personal voice assistants (PVA)

Participants indicated their attitudes towards PVAs on three semantic differential items (with five gradation points) that were created for the purpose of this study (e.g., “hate it—love it”; Cronbach’s α = 0.94, *M* = 3.72, *SD* = 1.09; see Supplement S3).

##### Attitudes towards robots

People’s general assessment of robots was measured with three items that were previously used in robot acceptance research^24^. The items were answered on a four-point scale (Cronbach’s α = 0.77, *M* = 3.24, *SD* = 0.57; e.g., “Robots are a good thing for societies because they help people”, with 0 = *totally disagree* to 3 = *totally agree*, see Supplement S3).

##### Social desirability

Participants’ tendency to give socially desirable answers was measured with the Social Desirability Scale^53^. In this measure, participants have to answer whether 16 socially desirable or undesirable actions match their own behavior on a dichotomous scale (“true” or “false”). The number of socially desirable responses is added up for each participant, resulting in a range between 0 and 16 (Cronbach’s α = 0.85, *M* = 8.74, *SD* = 0.83).

### Results

Our analysis of factorial validity was guided by the assumption that the items represent a single construct: People’s attitude towards AI. To this end, we compared different, increasingly less restrictive, models in a confirmatory factor analysis: The first model (a) specified a single factor and, thus, assumed that individual differences in item responses could be explained by a single, general attitude construct. However, this assumption is often too strong in practice because specific content facets or item wording might lead to minor multidimensionality. Therefore, Model (b) estimated a bifactor S-1 structure that, in addition to the general factor, included two orthogonal specific factors for the cognitive or affective items. These specific factors captured the unique variance resulting from the two content domains that were not accounted by the general attitude factor. Following Eid and colleagues^54^, no specific factor was specified for the behavioral items which, thus, acted as reference domain. Because the ATTARI-12 included positively and negatively worded items, Model (c) explored potential wording effects by evaluating a bifactor S-1 model that included an orthogonal specific factor for the negatively worded items. Finally, we combined Models (b) and (c) to study the joint effects of content and method effects.

The goodness of fit of these models are summarized in Table 2. Using established recommendations for the interpretation of these fit indices^55^, the single factor model exhibited an inferior fit to the data. Also, acknowledging the different content facets (cognitive, affective, behavioral) did not improve model fit. However, the ATTARI-12 exhibited non-negligible wording effects as demonstrated by Model (c). This model showed a good fit to the data with a comparative fit index (CFI) of 0.98, a root mean squared error (RMSEA) of 0.03, and a standardized root mean residual (SRMR) of 0.03. Despite the observed multidimensionality, all items showed good to excellent associations with the general factor as evidenced by rather high factor loadings falling between 0.48 and 0.88 (see Table 3). To examine the consequence of the multidimensional measurement structure in more detail, we examined how much of the systematic variance in the ATTARI-12 was explained by the general factor or the specific wording factor (cf.^56^). While the wording factor explained about 21% of the variance, most of the common variance (79%) was reflected by the general factor. As a result, the hierarchical omega reliability for the general factor was ω_*H*_ = 0.83 but only ω_*H*_ = 0.38 for the specific factor. These results emphasize that all twelve items comprised an essentially unidimensional scale operationalizing a strong general factor reflecting the overarching attitude towards AI. Based on the analyses, no items needed to be excluded from the scale due to subpar psychometric properties.

**Table 2:** Goodness of fit for competing confirmatory factor models for the ATTARI-12 (Study 1; US-American MTurk Panelists).

		χ^2^	*df*	CFI	RMSEA	SRMR	AIC	BIC	Comp	Δχ^2^	Δ*df*	*p*
(a)	Single factor model	434.77	54	0.85	0.15	0.08	14,564	14,664				
Bifactor S-1 models with one global factor and orthogonal specific factors for …												
(b)	Content facets	327.39	46	0.89	0.14	0.07	14,412	1456	(a)	102.95	8	< 0.001
(c)	Item wording	109.64	48	0.98	0.06	0.03	14,086	14,212	(a)	268.35	6	< 0.001
(d)	Content facets and item wording	93.91	40	0.98	0.06	0.03	14,076	14,236	(b)	191.45	6	< 0.001
									(c)	16.07	8	0.041

χ^2^ = Robust test statistic^83^, *df* degrees of freedom, *CFI* comparative fit index, *RMSEA* root mean squared error of approximation, *SRMR* standardized root mean residual, *AIC* Akaike’s information criterion, *BIC* Bayesian information criterion, *Comp.* Comparison model, *Δχ*^*2*^ Scaled chi square difference test statistic^84^.

**Table 3:** Factor loading pattern for the ATTARI-12 (Study 1; US-American MTurk panelists).

Item	Loading on general factor	Loading on specific factor	Residual variance
1	0.79 (0.04)/0.84		0.26
2	0.66 (0.05) /0.59	0.61 (0.06) /0.54	0.46
3	0.81 (0.04) /0.77		0.45
4	0.66 (0.05) /0.57	0.54 (0.06) /0.48	0.58
5	0.91 (0.04) /0.87		0.27
6	0.64 (0.05) /0.65		0.55
7	0.77 (0.05) /0.64	0.56 (0.06) /0.46	0.56
8	0.55 (0.05) /0.48	0.52 (0.06) /0.45	0.75
9	0.71 (0.05) / 0.65		0.69
10	0.67 (0.05) / 0.59	0.70 (0.06) / 0.61	0.37
11	0.95 (0.04) / 0.88		0.27
12	0.84 (0.05) / 0.69	0.59 (0.06) / 0.48	0.43

Presented are unstandardized factor loadings (with standard errors in parenthesis)/standardized factor loadings and residual variances. The latent factors were identified by constraining the latent factor variances to 1.

**Figure 1:**
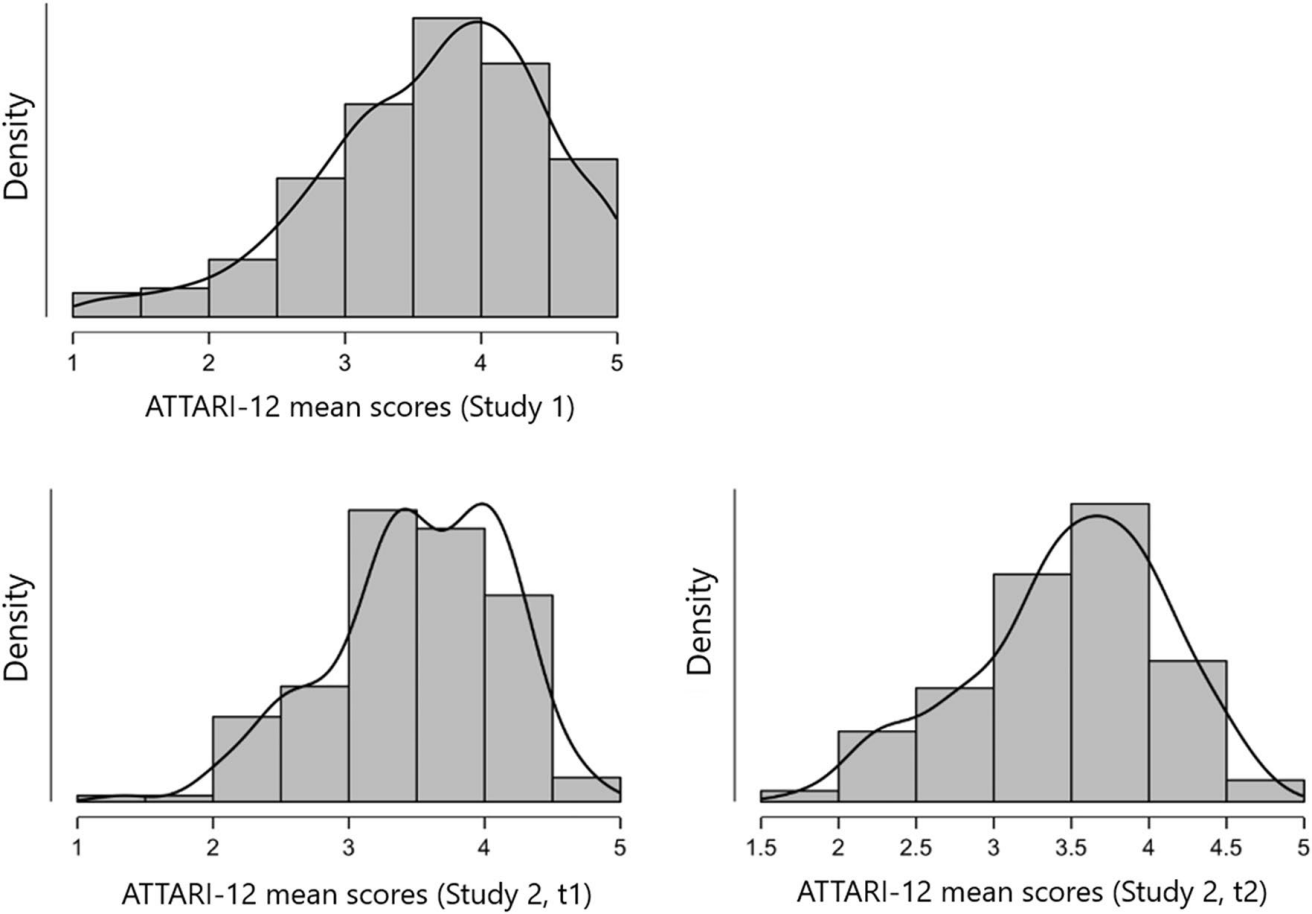
Distributions of the ATTARI-12 Results in Study 1 and Study 2 (at Time 1 and Time 2).

Descriptive and correlational results on the composite measure of the ATTARI-12 are shown in Table 4. The reliability in terms of internal consistency was excellent, Cronbach’s α = 0.93. The distribution approximated a normal distribution, skewness (= − 0.63) and kurtosis (= 0.22) were within the boundaries expected for psychometrically tested scales (also see Fig. 1). We acknowledge, however, that the distribution was somewhat left-skewed. As expected, the measure was positively correlated with attitudes towards electronic personal assistants (*r* = 0.60, *p* < 0.001) and with attitudes towards robots (*r* = 0.68, *p* < 0.001). ATTARI-12 scores were not substantially related to social desirability bias (*r* = 0.04, *p* = 0.411).

**Table 4:** Descriptive statistics and zero-order correlations (Study 1; US-American MTurk panelists).

							1	2	3	4	5
		Cronbach’s α	*M*	*SD*	Skew	Kurt					
1	ATTARI-12	0.93	3.66	0.83	− 0.63	0.22	−				
2	Social Desirability	0.85	8.74	4.24	− 0.25	− 0.87	0.037	–			
3	Attitude Towards PVAs	0.94	3.72	1.09	− 0.81	0.02	0.599***	0.125**	–		
4	Attitude Towards Robots	0.77	3.24	0.57	− 0.75	0.81	0.676***	− 0.021	0.435***		
5	Age		39.78	11.06	0.81	− 0.08	− 0.039	0.090*	− 0.023	− 0.036	
6	Gender						− 0.087	0.042	0.099*	− 0.058	0.164***

*N* = 485 for analyses that involve gender, *N* = 490 for all other analyses. *p < 0.05, **p < 0.01, ***p < 0.001. Gender was dummy-coded (0 = male; 1 = female). PVAs = Personal Voice Assistants.

In sum, the one-dimensional ATTARI-12 measured attitudes towards AI in a reliable way, and the indicators of convergent (attitudes towards specific AI applications) and divergent (social desirability) construct validity corroborate the validity of the scale.

## Study 2

The aim of the second study was to develop a German-language version of the scale and to gain further insight into the scale’s reliability and validity. In particular, we examined the re-test reliability of the scale, by administering the items at two points in time. We further assessed the extent to which the participants (undergraduates) wished to work with (or without) AI in their future careers. We expected a positive association between the latter variable and attitudes towards AI.

### Method

This study consisted of two parts, administered online with an average delay of 31.40 days (SD = 2.31; range 26–36 days). Students of a social science major at the University of Würzburg, Germany participated for course credit. According to calculations with G*Power software—based on an expected small to moderate correlation of *r* = 0.25, 80% power, and α = 0.05—a minimum sample size of 123 participants was required. Yet, as we expected dropouts from Time 1 to Time 2, a starting sample of 180 participants was targeted. Eventually, 166 participants completed the survey at Time 1, and a total of 150 students provided data at both points in time. This final sample of 150 participants with complete data had an average age of 21.21 years (*SD* = 2.60; range: 18 to 41 years) and consisted predominantly of women (113 female, 36 male, 1 other). Nearly all of the participants were German native speakers (see Supplement S4 for detailed descriptive data and Supplement S8 for the German-language version of the scale). Importantly, since participants for Study 2 were recruited among German students (in contrast to the use of English-language survey panel members in Study 1), we are confident that our two validation studies were based on entirely different samples.

At time 1, participants answered the ATTARI-12, as well as four items that measured their interest in a career involving AI technology (two items were reverse-coded, e.g., “I would prefer a position in which AI plays no role”). The items went with a 5-point scale (1 = *strongly disagree*, 5 = *strongly agree*), Cronbach’s α = 0.85 (see Supplement S5). In the resulting index, higher scores indicated higher aspirations for AI-related careers. At the study’s second measurement time, the ATTARI-12 had to be answered once again. Furthermore, the study was initially designed to also include a measure of social desirability at this point (^53^, in its German translation); however, the respective assessment did not achieve satisfactory reliability (Cronbach’s α < 0.40) and was, therefore, excluded from our analysis.

### Results

The main results are shown in Table 5. The reliability in terms of internal consistency was very good for the German-language version applied in this study, T1 Cronbach’s α = 0.91; T2 Cronbach’s α = 0.89. The distribution approximated a normal distribution, skewness (skewness T1 = − 0.58; skewness T2 = − 0.48) and kurtosis (kurtosis T1 = 0.07; kurtosis T2 = − 0.24) were within the boundaries expected for psychometrically sound scales (Fig. 1). Importantly, the test-re-test association was large, *r*(148) = 0.804, *p* < 0.001, supporting the reliability of the scale. Associations with the AI-career measure were significant and ranged around *r* = 0.60. In sum, the one-dimensional ATTARI-12 (German version) showed good psychometric properties.

**Table 5:** Descriptives and zero-order correlations (Study 2; German University Students).

						ATTARI-12 T1	ATTARI-12 T2
	Cronbach’s α	*M*	*SD*	Skew	Kurt		
ATTARI-12 T1	0.90	3.51	0.64	− 0.58	0.07	–	
ATTARI-12 T2	0.89	3.49	0.63	− 0.48	− 0.24	**0.804**	–
Career-AI T1	0.85	3.11	0.92	− 0.08	− 0.78	0.635	0.563

*N* = 166 for both variables assessed at Time 1 (T1). *N* = 163 for ATTARI-12 assessed at Time 2 (T2). *N* = 150 for the correlations between T1- and T2-variables. All correlations are significant at *p* < 0.001 (two-tailed). The value printed in bold font represents the focal re-test reliability.

## Study 3

Having found empirical support for the validity of our newly created measure, we proceeded with our second main research goal: Exploring potential associations between personality traits and attitudes towards AI. To ensure transparency, we decided to preregister Study 3 before data collection, including all hypotheses and planned analyses (https://aspredicted.org/VRU_BJI).

### Method

#### Participants

An a priori calculation of minimum sample size by means of G*Power software (assuming a small to moderate effect of *f*^2^ = 0.08, 80% power, α = 0.05, and eleven predictors in a hierarchical linear regression) resulted in a lower threshold of 221 participants. Since we intended to screen our sample for several data quality indicators and, thus, desired some leeway for potential exclusions, we recruited 353 US-American participants via the Amazon MTurk participant pool (age: *M* = 38.34 years, *SD* = 10.93; 112 female, 239 male, 2 other; payment $1.50). Specifically, we used the following MTurk criteria to ensure high data quality^57,58^: (a) at least 100 approved HITs; (b) HIT approval rate > 97%. Moreover, the web service “IPhub.info” was used to check whether users’ individual IP address correctly indicated the United States as a current location.

Examining the collected data, several measures were taken to exclude MTurk workers whose answers indicated careless and inattentive responding. First, we asked all participants to indicate their age and year of birth on two separate pages of the survey; if these answers deviated more than two years from each other, the participant was excluded (*n* = 10). Second, as a specific check against bot workers, participants were asked to name a type of vegetable (“eggplant”) that was depicted on a large-scale photograph. Apart from minor typos, all answers that did not resemble the correct answer in English language led to the removal of the data (*n* = 6). Third, participants were asked to choose the correct study topic (“artificial intelligence”) in a multiple-choice item, a task that was not fulfilled correctly by another *n* = 9 participants.

In addition, we initially considered removing all participants who had filled in the survey in less than four minutes, a threshold we had measured as the minimum time for attentive responding. Looking at our obtained data, this would have led to the additional exclusion of 81 participants. However, as we noticed that a lot of MTurk workers had finished the questionnaire in slightly less than four minutes, we decided to ease our initial exclusion criterion to a minimum duration of three minutes—leading to the exclusion of *n* = 30 participants. To make sure that this deviation from our preregistered analysis plan would not substantially change our results, we repeated all planned analyses with the initial four-minute criterion. Doing so, we found no noteworthy statistical differences (the results of this additional analysis are presented in Supplement S6).

Thus, in summary, our final sample consisted of 298 participants with a mean age of 39.29 years (*SD* = 11.08; range from 22 to 73 years). Gender balance was slightly skewed towards men (102 female, 195 male, 1 other). Regarding ethnicity, most participants identified themselves as White (77.9%), followed by Asian (8.1%), Black (7.7%), and Hispanic (3.7%). Level of education was relatively high, with most participants having obtained either a bachelor’s degree (49.7%), a master’s degree (16.1%) or a Ph.D. (2.7%). Lastly, we note that our sample was quite balanced in terms of political orientation. For a complete overview of the obtained descriptive data, please consult Supplement S7.

#### Measures

All items of the following measures were presented on 5-point scales (1 = *strongly disagree*; 5 = *strongly agree*).

##### Attitudes towards AI

To measure the main outcome variable, we administered the ATTARI-12 scale in its English version. Our data analysis showed that the scale again yielded excellent internal consistency this time around (Cronbach’s α = 0.92).

##### Big Five

The Big Five were assessed with the Big Five Inventory^59^, which consists of 44 items (openness: ten items, e.g., “I am a someone who is curious about many different things”; conscientiousness: nine items, e.g., “…does a thorough job”; extraversion: 8 items, e.g., “…is talkative”; agreeableness: nine items, e.g., “…is considerate and kind to almost everyone”; neuroticism: 8 items, e.g., “…can be tense”). We observed very good to excellent internal consistency for all five measured dimensions (openness: Cronbach’s α = 0.85; conscientiousness: Cronbach’s α = 0.85; extraversion: Cronbach’s α = 0.90; agreeableness: Cronbach’s α = 0.81; neuroticism: Cronbach’s α = 0.90).

#### Dark Triad

We assessed participants’ Dark Triad personality traits with the Short Dark Triad scale (SD3^60^). This instrument includes nine items on Machiavellianism (e.g., “It’s not wise to tell your secrets”), six items on psychopathy (e.g., “People often say I’m out of control.”), and nine items on narcissism (e.g., “Many group activities tend to be dull without me.”). Reliability analyses suggested good to very good internal consistencies for all three scales (Machiavellianism: Cronbach’s α = 0.84; psychopathy: Cronbach’s α = 0.83; narcissism: Cronbach’s α = 0.78).

#### Conspiracy mentality

The Conspiracy Mentality Questionnaire (CMQ^33^) offers a measure of people’s general susceptibility to conspiracy theories and beliefs. It consists of five items that encapsulate an inherent skepticism about the workings of society, governments, and secret organizations (e.g., “I think that many important things happen in the world, which the public is never informed about.”; “I think that events which superficially seem to lack a connection are often the result of secret activities.”). With our data, we observed a very good Cronbach’s α of 0.86 for the CMQ.

### Results

Table 6 summarizes descriptive information for and zero-order correlations between all study variables. On average, participants’ AI-related attitudes were slightly positive, *M* = 3.60, *SD* = 0.81—considering that a value of 3 indicates the neutral midpoint of our 5-point scale, which was assembled from equal numbers of negative (inversed) and positive items.

**Table 6:** Descriptive statistics and correlations for study variables (Study 3; US-American MTurk Panelists).

	*Variable*	*M*	*SD*	1	2	3	4	5	6	7	8	9	10	11	12
1	Age	39.29	11.08	–											
2	Gender^1^			− 0.23***	–										
3	ATTARI-12	3.60	0.81	− 0.12*	0.04	–									
4	Openness to Experience	3.56	0.74	0.02	0.01	0.14*	–								
5	Conscientiousness	3.97	0.72	0.25***	− 0.12*	0.08	0.20***	–							
6	Extraversion	2.77	1.00	0.04	0.07	0.04	0.27***	0.25***	–						
7	Agreeableness	3.77	0.73	0.19***	− 0.15**	0.22***	0.21***	0.54***	0.16**	–					
8	Neuroticism	2.64	1.01	− 0.13*	− 0.10	− 0.07	− 0.14*	− 0.60***	− 0.44***	− 0.53***	–				
9	Machiavellianism	2.88	0.81	− 0.19**	0.17**	− 0.11	− 0.05	− 0.25***	0.07	− 0.50***	0.25***	–			
10	Psychopathy	2.19	0.77	− 0.25***	0.30***	− 0.12*	− 0.02	− 0.36***	0.27***	− 0.50***	0.14*	0.68***	–		
11	Narcissism	2.50	0.74	− 0.15**	0.20***	− 0.06	0.23**	− 0.03	0.59***	− 0.18**	− 0.16**	0.48***	0.62***	–	
12	Conspiracy Mentality	3.36	0.94	− 0.08	− 0.06	− 0.22***	0.06	− 0.07	0.07	− 0.15**	0.15*	0.33***	0.26***	0.26***	–

*Note*. *N* = 298. * p < 0.05, ** p < 0.01. ^**1**^Gender coded with “0” = female, “1” = male.

With our main data analysis slated to involve multiple regression, we first made sure that all necessary assumptions were met^61^: Residuals were independent and normally distributed, and neither multicollinearity nor heteroskedascity issues could be found. Moreover, satisfying Cook’s distance values revealed that no influential cases were biasing our model. As such, we proceeded with hierarchical linear regression as the core procedure of our data analysis. Using participants’ ATTARI score as the criterion, we first entered their age and gender as predictors (Step 1), before adding the Big Five (Step 2), the Dark Triad (Step 3), and conspiracy mentality (Step 4) to an extended regression model. Table 7 presents the main calculations and coefficients of this hierarchical regression analysis. As can be seen here, the first step of the regression resulted in an insignificant equation, *F*(2, 294) = 2.18, *p* = 0.115, with an *R*^*2*^ of 0.02. In contrast to this, the second step of the procedure (including the Big Five) yielded a significant result, *F*(7, 289) = 4.10, *p* < 0.001, Δ*R*^*2*^ = 0.08. Proceeding with Step 3, we found that adding the Dark Triad traits did not lead to a significant increase of *R*^*2*^ (Δ*R*^*2*^ < 0.01, *p* = 0.52). Entering participants’ conspiracy mentality as a final predictor, however, Step 4 resulted in a solution with significantly higher explained variance, *F*(11,285) = 4.17, *p* < 0.001, *R*^*2*^ = 0.14 (Δ*R*^*2*^ = 0.04). In the following, we will take a closer look at the predictive value of each entered predictor.

**Table 7:** Hierarchical regression predicting participants’ attitudes towards AI (Study 3).

	Step 1		Step 2		Step 3		Step 4	
	β	*t*	β	*t*	β	*t*	β	*t*
Age	− 0.12	− 1.96	− 0.15*	− 2.52	− 0.16**	− 2.68	− 0.17**	− 2.86
Gender^1^	0.01	0.24	0.06	0.95	0.07	1.15	0.04	0.68
Openness to experience			0.09	1.53	0.10	1.67	0.11	1.85
Conscientiousness			− 0.01	0.09	− 0.03	− 0.35	− 0.01	− 0.15
Extraversion			0.01	0.08	0.08	0.93	0.07	0.85
Agreeableness			0.29***	3.95	0.25**	3.12	0.27***	3.34
Neuroticism			0.08	1.01	0.07	0.81	0.10	1.24
Machiavellianism					0.04	0.48	0.09	1.08
Psychopathy					− 0.06	− 0.64	− 0.05	− 0.49
Narcissism					− 0.09	− 1.02	− 0.05	− 0.60
Conspiracy mentality							− 0.22***	− 3.68
*R*^*2*^ (Δ*R*^*2*^)	0.02		(0.08***)		(0.01)		(0.04***)	

*N* = 298. *p < 0.05, **p < 0.01, ***p < 0.001. ^**1**^ Gender coded with “0” = female, “1” = male.

#### Predictive value of the Big Five

In response to hypotheses H1 through H5, we first directed our attention to the Big Five personality dimensions. Based on the second regression step, which was designed to include these variables, it was found that only agreeableness significantly predicted participants’ attitudes towards AI (*p* < 0.001), with a positive beta coefficient of 0.29. This positive association also persisted after entering other personality traits in Steps 3 and 4 of the regression. Hence, we conclude that higher agreeableness was related to more positive views about AI technology in our sample—providing empirical support for Hypothesis 4. Out of the other Big Five dimensions, openness to experience slightly missed the threshold of statistical significance in the final regression step, β = 0.11, *p* = 0.065, so that further exploration of the respective hypothesis may be warranted before rejecting it entirely. The remaining Big Five traits conscientiousness (H2), extraversion (H3), and neuroticism (H5), however, remained clearly insignificant as predictors.

#### Predictive value of the Dark Triad

Proceeding to the dark personality traits Machiavellianism, psychopathy, and narcissism (as entered during Step 3), we note that none of the three predictors approached the conventional threshold of statistical significance. As such, we cannot accept hypotheses H6 through H8; according to our data, more malevolent character traits were not predictive of people’s general attitudes about AI.

#### Predictive value of conspiracy mentality

Concluding our investigation of dispositional influences, we focused on participants’ conspiracy mentality as it was added during the regression’s final step. We found that this variable emerged as another meaningful predictor, β = − 0.22, *p* < 0.001. In support of Hypothesis 9, we therefore report that a stronger conspiracy mentality was related to more negative views towards artificially intelligent technology.

#### Predictive value of age and gender

Lastly, the impact of participants’ age and gender on their AI attitudes was explored. To this end, we focused on our final regression model with all predictors entered into the equation. While the influence of gender remained insignificant, it was examined that higher age was significantly related to lower scores in the ATTARI-12 scale, i.e., to more aversive cognitions, feelings, and behavioral intentions towards AI (β = − 0.17, *p* = 0.005). In light of this, our data successfully replicated previous findings regarding this sociodemographic variable, resulting in a positive answer to H11. Meanwhile, we reject H10 on the influence of gender.

## General discussion

In a world shaped by autonomous technologies that are supposed to make human life safer, healthier, and more convenient, it is important to understand how people evaluate the very notion of artificially intelligent technology—and to identify factors that account for notable interindividual variance in this regard. Thus, the current project set out to explore the role of fundamental personality traits as predictors for people’s AI-related attitudes. In order to build our research upon a valid and reliable measurement of the outcome in question, we first developed a novel questionnaire—the ATTARI-12—and confirmed its psychometric quality across two studies. Distinguishing our instrument from extant alternatives, we note that our one-dimensional scale assesses attitudes towards AI on a full spectrum between aversion and enthusiasm. Furthermore, the ATTARI-12 items incorporate the classic trichotomy of human attitude (cognition, emotion, behavior), thus emerging as a conceptually sound way to measure people’s evaluation of AI. Lastly, since the instruction of the ATTARI-12 does not focus on specific use cases but rather on a broader understanding of AI as a set of technological abilities, we believe it may be suitable to be used across many different disciplines and research settings.

Utilizing our newly developed measure, we proceeded to our second research aim: Investigating potential connections between individuals’ AI-related attitudes and their personality traits. Specifically, we focused on two central taxonomies from the field of personality psychology (the Big Five, the Dark Triad), as well as conspiracy mentality as a trait of high contemporary relevance. By these means, we found significant effects for two of the explored dispositional predictors: Agreeableness (one of the Big Five traits) was significantly related to more positive attitudes about AI, whereas stronger conspiracy mentality predicted the opposite. We believe that both of these findings may offer intriguing implications for researchers, developers, and users of autonomous technology.

A personality dimension that typically goes along with an optimistic outlook at life^62,63^, agreeableness has been found to predict people’s acceptance of innovations in many different domains^64^. Psychologically speaking, this makes perfect sense: In line with their own benevolent nature, agreeable individuals tend to perceive others more positively as well—and may therefore come to think more about the opportunities than the risks of a new invention when forming their opinion. Since our specific measurement of participants’ attitudes requested them to consider both positive and negative aspects of AI, this tendency to focus more on the upside might have played an important role for the observed effect. Furthermore, we suppose that the trusting nature that often characterizes agreeable individuals^65^ offers another reasonable explanation for our result. Faced with a complex concept such as AI, which may appear obscure or downright incomprehensible to the layperson user, it arguably becomes all the more important for people to understand the intentions of the decision makers and stakeholders behind it. While the technological companies that develop AI systems might not always inspire the necessary confidence with their actions (e.g., excessive data collection, convoluted company policies), having a stronger inclination to trust others will likely compensate for this—presenting another reason as to why agreeableness might have emerged as a significant predictor in the current study.

A surprisingly similar argument may be put forward when addressing the second dispositional factor that achieved notable significance in our analyses, i.e., people’s conspiracy mentality. By definition, this personality trait is also anchored in perceptions of trust (or rather, a lack thereof), a notion that is echoed by our findings: The stronger participants expressed their skepticism about governments, organizations, and related news coverage (as indicated by items such as “I think many very important things happen in the world, which the public is never informed about”), the more negatively did their attitudes about AI turn out. In our reading, this further illustrates how transparency and perceived trustworthiness crucially affect the public acceptance of AI; just as having an agreeable, credulous character predicted more favorable attitudes, the disposition to suspect sinister forces on the global stage related to more negative views about autonomous technology.

On a societal level, we believe that our identification of conspiracy beliefs as a pitfall for AI-related attitudes is a particularly timely result, as it connects to the on-going debate about *fake news* in modern society. In the past few years, many people have started turning to social media as a source for news and education, which has paved the way for an unusual proliferation of misinformation^66^. Virtual spaces, in which like-minded individuals mutually confirm their attitudes and suspicious about different issues (so-called *echo chambers*; e.g., discussion forums or text messaging groups) have become highly prevalent, and, by these means, turned into a cause for concern among media scholars. Indeed, research suggests that using platforms such as Facebook, Twitter, or YouTube not only provides an immensely effective way to disseminate postfactual beliefs but may even increase people’s susceptibility to conspiracy theories in the first place^67^. Taken together with the fact that it is mostly complex and ambiguous topics for which people seek out postfactual information^68^, negative views about intelligent technology may proliferate most easily in the online realm. Arguably, the much-observed social media claim that COVID-19 vaccines secretly inject AI nanotechnology into unwilling patients^45^ illustrates this perfectly.

Based on the presented arguments, it stands to reason that the successful mass-adoption of intelligent technologies may also depend on whether postfactual theories about AI can be publicly refuted. In order to do so, it might be key to increase the public’s trust, not only in the concept of AI itself, but also in the organizations and systems employing it. For instance, developers may set out to educate users about the capabilities and limits of autonomous technologies in a comprehensible way, whereas companies might strive for more accessible policies and disclosures. At the same time, we note that overcoming deep-rooted fears and suspicions about AI will likely remain a great challenge in the future; in our expectation, the sheer sophistication and complexity of intelligent computers will continue to offer fertile ground for conspiracy theorists. Also, it cannot be ruled out that attempts to make AI more transparent in the future could also backfire, as conspiracy theorists might interpret these attempts as actual proof for a conspiracy in the first place. More so, the fact that popular movies and TV shows frequently present AI-based technology in a dangerous or creepy manner (e.g., in dystopian science fiction media^23^) may make it even more difficult to establish sufficient levels of trust—especially among those who tend to have a more skeptical mentality to begin with.

Apart from the interesting implications offered by the two significant personality predictors, several other examined traits showed no significant association with participants’ AI-related attitudes. Specifically, we found that three of the Big Five (conscientiousness, extraversion, and neuroticism) and all Dark Triad variables fell short in explaining notable variance in the obtained ATTARI-12 scores. Taken together with the abovementioned results, this indicates that user personality may have valuable yet limited potential to explain public acceptance of AI technology. In their nature as overarching meta-level traits, certain Big Five dimensions might simply be too abstract to address the nuances that characterize people’s experiences with—and attitudes towards—AI technology. For example, being a more extraverted person might involve aspects that both increase acceptance of AI (e.g., by being less apprehensive and restrained in general) and decrease it (e.g., by valuing genuine social connection to other humans, which may be challenged by new AI technology). Similarly, the more deviant personality traits included in the Dark Triad might be connected to both positive and negative views towards AI, thus preventing a significant prediction in a specific direction. Just as the many possibilities brought by new intelligent technology could be seen as useful tools to act manipulatively or enhance the self—suggesting a positive link to Machiavellianism and narcissism—they may also raise concerns among people scoring high in these traits, as AI might make it harder to act out devious impulses in an undetected or well-accepted manner.

### Limitations and future directions

We would like to point out important limitations that need to be considered when interpreting the presented results of our project, especially the third and final study that served to address our main hypotheses. Although we recruited a relatively diverse sample in terms of age, gender, and socioeconomic background—and put several measures into place to ensure high data quality—our data on the association between AI attitudes and user personality are based on only one group of participants. Moreover, given that the sample of our third study was recruited via the same method as used in Study 1 (MTurk), we cannot rule out that certain participants might have taken part in both research efforts (even though, based on the very large participant pool of the chosen panel, we deem it highly unlikely that duplicate responses occurred). As such, replication efforts with different samples are encouraged in order to consolidate the yielded evidence. Not least, this concerns the recruitment of samples with a more balanced gender distribution, as our own recruitment yielded a notable majority of male participants. Also, considering that socio-economic and educational factors exert notable impact on people’s opinions towards technology (not least regarding AI; see^6^), shifting focus to different backgrounds should be most enlightening regarding the generalizability of our work. We believe that this might be especially important when focusing further on the dimension of conspiracy mentality—keeping in mind that a lack of education, media literacy, and analytic thinking ability have all been shown to predict the susceptibility to post-factual information^43,69,70^.

Along these lines, we suggest that studies with samples from different cultures should be carried out in order to establish whether our findings are consistent across national borders. Most recently, a study focusing on a sample of South Korean participants also reported positive associations between attitudes towards AI and the Big Five dimension agreeableness, especially regarding the sociality and functionality of AI-powered systems^71^. In contrast to our research, however, the authors did not pursue a one-dimensional measurement, thus yielding ambivalent findings (e.g., conscientiousness related to negative emotions towards AI but also to expectations of high functionality). Hence, we consider it worthwhile to apply the one-dimensional ATTARI-12 in other cultures in order to better understand people’s stance towards AI—and how this attitude is shaped by certain personality traits. Keeping in mind that our newly developed scale facilitated a reliable, one-dimensional measurement with samples in two countries, it appears as a promising tool for such examinations; even though, of course, further validation of our instrument is clearly welcome, especially for its non-English versions. Moreover, intercultural comparisons will have to ensure a consistent measurement of the personality traits in question. While both the Big Five and the Dark Triad have been highlighted for their cultural invariance^72,73^, some studies have also raised doubts about this claim^74^—so that the applicability of the used personality taxonomies might pose an additional challenge in this regard. A potential solution here would be to use the HEXACO inventory of personality^75^, which combines both the Big Five and the Dark Triad’s deviant traits into a novel taxonomy of human personality. Not only has the HEXACO model received strong support in terms of cultural invariance^76^, using it would also enable scholars to cross-validate the findings of the current study with another well-established instrument. Of course, completely different personality traits than the ones included in our endeavor—e.g., impulsivity, sensation seeking, or fear of missing out—could also be helpful to gain a thorough understanding of interindividual differences regarding people’s attitudes towards AI.

Additionally, it should be noted that future research into this topic can clearly benefit from assessing situational and motivational aspects that affect participants’ views on AI beyond their personality characteristics. For instance, recent research suggests that views on sophisticated technology are strongly modulated by prior exposure to science fiction media^23,77^, as well as philosophical views and moral norms^78^. Furthermore, people have been found to change their attitudes after several encounters with AI technology ^79^, adding another factor to the equation. Taken together, this suggests that future studies might tap into several profound covariates to disentangle the states and traits affecting attitudes towards AI. In any case, keeping in mind that our newly developed ATTARI-12 scale facilitated a reliable, one-dimensional measurement with samples of different age ranges and cultural backgrounds, we suggest that it constitutes a promising methodological cornerstone for future examinations of AI attitudes.

## Conclusion

In our project, we investigated attitudes towards AI as a composite measure of people’s thoughts, feelings, and behavioral intentions. Striving to complement previous research that focused more on the acceptance of specialized AI applications, we created and utilized a new unidimensional measure: the ATTARI-12. We are confident that the developed measure may now serve as a useful tool to practically address AI as a macro-level phenomenon, which—despite encompassing a host of different programs and applications—is united by several shared fundamentals. In our expectation, the created scale can still be administered even when new and unforeseen AI technologies emerge, as it focuses more on underlying principles than specific capabilities. For practitioners and developers, this perspective may be particularly insightful, suggesting that individuals’ responses to new AI technology (e.g., as customers or employees) will not only be driven by the specific features of a certain technology, but also by a general attitude towards AI. Similarly, we hope that large-scale sociodemographic efforts (e.g., national opinion compasses) might be able to benefit from the provided measure.

Apart from that, we established significant connections between general AI attitudes and two selected personality traits. In our opinion, it is especially the observed association with participants’ conspiracy mentality that holds notable relevance in our increasingly complex world. If AI developers cannot find suitable ways to make their technology appear innocuous to observers (in particular to those who tend to seek out post-factual explanations), it might become increasingly challenging to establish innovations on a larger scale. However, we emphasize that negative attitudes and objections against AI technology are not necessarily unjustified; hence, when educating the public on AI technologies, people should be encouraged to reflect upon both potential risks and benefits. To reduce the risk for new conspiracy theories, we further encourage industry professionals to stay as transparent as possible when introducing their innovations to the public.

Lastly, we suggest that follow-up work is all but needed to elaborate upon our current contribution. While we remain convinced that measuring attitudes towards AI as a general set of technological abilities is meritorious, more tangible results could stem from including a theoretical dichotomy that has recently emerged in the field of human–machine interaction^80–82^. More specifically, it might be worthwhile to distinguish between AI abilities that relate to agency (i.e., planning, thinking, and acting) and those that relate to experience (i.e., sensing and feeling). In turn, scholars may be able to find out whether different user traits also relate to different attitudes towards “acting” and “feeling” AI—a nuanced perspective that would still allow for broader implications across many different technological contexts.

### Supplementary Information

Supplementary Information.

## Data Availability

All materials, obtained data, and analysis codes for the three reported studies can be found in the project’s Open Science Framework repository (https://osf.io/3j67a/).

## Appendix

### Attitudes towards artificial intelligence Scale (ATTARI-12), English version

**Instruction:** In the following, we are interested in your attitudes towards artificial intelligence (AI). AI can execute tasks that typically require human intelligence. It enables machines to sense, act, learn, and adapt in an autonomous, human-like way. AI may be part of a computer or online platform—but it can also be encountered in various other hardware devices such as robots.

Item List:

**Table:**

	Wording	Facet	Valence
1	*AI* will make this world a better place.	*Cognitive*	Positive
2	I have strong negative emotions about *AI*.	*Affective*	Negative (reverse-coded)
3	I want to use technologies that rely on *AI*.	*Behavioral*	Positive
4	*AI* has more disadvantages than advantages.	*Cognitive*	Negative (reverse-coded)
5	I look forward to future *AI* developments.	*Affective*	Positive
6	*AI* offers solutions to many world problems.	*Cognitive*	Positive
7	I prefer technologies that do not feature *AI.*	*Behavioral*	Negative (reverse-coded)
8	I am afraid of *AI.*	*Affective*	Negative (reverse-coded)
9	I would rather choose a technology with *AI* than one without it.	*Behavioral*	Positive
10	*AI* creates problems rather than solving them.	*Cognitive*	Negative (reverse-coded)
11	When I think about *AI*, I have mostly positive feelings.	*Affective*	Positive
12	I would rather avoid technologies that are based on *AI.*	*Behavioral*	Negative (reverse-coded)
