# Supplementary material for: Attitudes towards AI: measurement and associations with personality
Source: Sci Rep. 2024 Feb 5;14:2909. doi: 10.1038/s41598-024-53335-2 (PMC10844202; doi:10.1038/s41598-024-53335-2)
Supplement: Supplementary file 1 — Supplementary Information. [file 41598_2024_53335_MOESM1_ESM.docx]

**Supplementary Materials**

for the manuscript “Attitudes Towards AI: Measurement and Associations with Personality”

**Supplement S1: Minor adjustments compared to the preregistration**

The information and procedure presented in the manuscript differs from the preregistration in two minor regards: First, we used different working titles for the ATTARI-12 in the preregistrations (Study 1 & 2: WATAI; Study 3: ATAIS-12). Yet, for reasons of clarity, the final acronym ATTARI-12 is chosen throughout the manuscript. Second, the preregistration of Study 1 stated that exclusion criteria would include an outlier analysis for completion time. The term *outlier analysis*, however, was not the ideal choice of words here. Instead, all participants who needed less than 120 seconds to complete the questionnaire were excluded from the analyses. This value was determined by inspecting the distribution of duration times—which, in our opinion, creates a more meaningful criterion than an outlier analysis that is merely based on standard deviations.

**Supplement S2: Descriptive Statistics for Study 1 (US-American MTurk Sample)**

| *Characteristic* | | *n* | % of sample |
| --- | --- | --- | --- |
| Gender | |  |  |
|  | Female | 212 | 43.3 % |
|  | Male | 273 | 55.7 % |
|  | Non-binary | 2 | 0.4 % |
|  | Prefer not to answer | 3 | 0.6 % |
| Education | |  |  |
|  | Some High School | 3 | 0.6 % |
|  | High School Graduate | 144 | 29.4 % |
|  | Bachelor’s Degree | 272 | 55.5 % |
|  | Master’s Degree | 58 | 11.8 % |
|  | Ph.D. or higher | 9 | 1.8 % |
|  | Trade School | 4 | 0.8 % |
| Ethnicity^1^ | |  |  |
|  | Black or African American | 40 | 8.2 % |
|  | Asian or Asian American | 34 | 6.9 % |
|  | Hispanic or Latino American | 25 | 5.1 % |
|  | Native American or Alaskan Native | 2 | 0.4 % |
|  | White | 405 | 82.7 % |
|  | Other | 3 | 0.6 % |
|  |  | *M* | *SD* |
| Age in years | | 39.78 | 11.06 |

*Notes*. *N* = 490. ^1^Participants could report more than one ethnicity.

**Supplement S3: Measures on Specific AI Applications (Study 1)**

***Measure for Attitudes towards Personal Voice Assistants***

**[Instruction]** Now we are interested in your opinion regarding personal voice assistants (e.g., Alexa, Echo, Siri or Bixby). Personal voice assistants may be embedded in phones, tablets, smart-speakers, or other small hardware devices. They are responsive to the user's voice and may react to a large number of vocal commands (such as “Start a call to Sarah” or “Dim the lights to 20%”).
Please indicate how you are feeling about personal voice assistants.

| hate it | ○ ○ ○ ○ ○ | love it |
| --- | --- | --- |
| negative | ○ ○ ○ ○ ○ | positive |
| repulsive | ○ ○ ○ ○ ○ | attractive |

***Measure for Attitudes towards Robots***

**[Instruction]** Now we are interested in your evaluation of robots. A robot is a machine which can assist humans in everyday tasks without constant guidance or instruction, e.g. as a kind of co-worker helping on the factory floor or as a robot cleaner, or in activities which may be dangerous for humans, like search and rescue in disasters.

**[Item 1]** Generally speaking, do you have a very positive, fairly positive, fairly negative or very negative view of robots? (0 = *very negative*, 3 = *very positive*)

**[Items 2 and 3]** Please indicate below how much you agree with the following statements. (0 = *totally disagree*, 3 = *totally agree*)

- Robots are a good thing for societies because they help people.
- Robots are necessary as they can do jobs that are too hard or too dangerous for people.

**Supplement S4: Descriptive Statistics for Study 2 (German Student Sample)**

| *Characteristic* | | *n* | % of sample |
| --- | --- | --- | --- |
| Gender | |  |  |
|  | Female | 113 | 75.3 % |
|  | Male | 36 | 24.0 % |
|  | Non-binary | 1 | 0.7 % |
| Profession | |  |  |
|  | College Student | 149 | 99.3 % |
|  | Employee or Self-Employed | 1 | 0.7 % |
| German Native Speaker | |  |  |
|  | Yes | 148 | 98.7 % |
|  | No | 2 | 1.3 % |
|  |  | *M* | *SD* |
| Age in years | | 21.21 | 2.60 |

*Notes*. *N* = 150. This table summarizes data for all participants who completed the survey at both measurement times.

**Supplement S5: Measure for Participants’ Interest in an AI-Related Career (Study 2)**

Note: This is an ad-hoc translation from German to English.

**[Instruction:]** Now we are interested in your future job choice. (1 = *completely disagree*, 5 = *completely agree*)

- I would like to encounter AI as part of my job.
- I would prefer a position in which AI plays no role. [reverse-coded]
- I would like to work with AI from time to time.
- Frequent contact with AI at the workplace would be deterring for me. [reverse-coded]

**Supplement S6**

*Hierarchical Regression Predicting Participants’ Attitudes Towards AI, Using the Stricter Cutoff in Terms of Minimum Completion Time (Study 3)*

|  | **Step 1** | |  | **Step 2** | |  | **Step 3** | | |  | **Step 4** | |
| --- | --- | --- | --- | --- | --- | --- | --- | --- | --- | --- | --- | --- |
|  | β | *t* |  | β | *t* |  | β | *t* | |  | β | *t* |
| Age | –.13^*^ | –2.05 |  | –.16^*^ | –2.33 |  | –.17^*^ | | –2.50 |  | –.18^***^ | –2.71 |
| Gender^1^ | .05 | 0.70 |  | .07 | 1.10 |  | .09 | | 1.24 |  | .05 | 0.77 |
| Openness to experience |  |  |  | .08 | 1.53 |  | .10 | | 1.67 |  | .11 | 1.60 |
| Conscientiousness |  |  |  | < .01 | 0.02 |  | < .01 | | < 0.01 |  | .01 | 0.13 |
| Extraversion |  |  |  | < .01 | < 0.01 |  | .08 | | 0.87 |  | .07 | 0.83 |
| Agreeableness |  |  |  | .21^***^ | 2.62 |  | .18^*^ | | 2.01 |  | .19^*^ | 2.18 |
| Neuroticism |  |  |  | .08 | 0.85 |  | .07 | | 0.72 |  | .10 | 1.08 |
| Machiavellianism |  |  |  |  |  |  | .01 | | 0.15 |  | .05 | 0.49 |
| Psychopathy |  |  |  |  |  |  | < .01 | | 0.02 |  | .02 | 0.15 |
| Narcissism |  |  |  |  |  |  | –.14 | | –1.36 |  | –.09 | –0.92 |
| Conspiracy mentality |  |  |  |  |  |  |  | |  |  | –.19^**^ | –2.80 |
| *F* | 2.87 | |  | 2.40^*^ | |  | 1.91^*^ | | |  | 2.50^***^ | |
| *R²* (Δ*R²*) | .02 | |  | (.04) | |  | (.01) | | |  | (.03^***^) | |
| *Note*. *N* = 247. **p* < .05, ***p* < .01, ****p* < .001. **^1^** Gender coded with “0” = female, “1” = male. | | | | | | | | | | | | |

**Supplement S7: Descriptive Statistics for Study 3 (US-American MTurk Sample)**

| *Characteristic* | | *n* | % of sample |
| --- | --- | --- | --- |
| Gender | |  |  |
|  | Female | 102 | 34.2 % |
|  | Male | 195 | 65.4 % |
|  | Other | 1 | 0.3 % |
| Education | |  |  |
|  | High School Graduate | 91 | 30.5 % |
|  | Bachelor’s Degree | 148 | 49.7 % |
|  | Master’s Degree | 48 | 16.1 % |
|  | Ph.D. or higher | 8 | 2.7 % |
|  | none of the above | 3 | 1.0 % |
| Ethnicity | |  |  |
|  | Black or African American | 23 | 7.7 % |
|  | Asian or Asian American | 24 | 8.1 % |
|  | Hispanic or Latino American | 11 | 3.7 % |
|  | Native American | 4 | 1.3 % |
|  | White | 232 | 77.9 % |
|  | Other | 4 | 1.3 % |
| Political Orientation | |  |  |
|  | Extremely Left | 39 | 13.1 % |
|  | Left | 93 | 31.2 % |
|  | Center | 65 | 21.8 % |
|  | Moderately Right | 72 | 24.2 % |
|  | Extremely Right | 29 | 9.7 % |
|  |  | *M* | *SD* |
| Age in years | | 39.29 | 11.08 |

*Notes*. *N* = 298.

**Supplement S8: Attitudes Towards Artificial Intelligence Scale (ATTARI-12), German version**

***Instruktion:*** *Im Folgenden interessieren wir uns für Ihre Einstellungen gegenüber Künstlicher Intelligenz (KI).* *Künstliche Intelligenz kann Aufgaben ausführen, die üblicherweise menschliche Intelligenz erfordern. Sie befähigt Maschinen dazu, selbstständig und ähnlich dem Menschen, ihre Umwelt wahrzunehmen, zu handeln, zu lernen und sich anzupassen. Künstliche Intelligenz kann Teil eines Computers oder einer Onlineplattform sein – man kann ihr aber auch in verschiedenen anderen technischen Geräten, wie etwa Robotern, begegnen.*

***Itemliste:***

|  | **Formulierung** | **Facette** | **Valenz** |
| --- | --- | --- | --- |
| **1** | Künstliche Intelligenz wird die Welt verbessern. | *Kognitiv* | Positiv |
| **2** | Ich habe starke negative Emotionen gegenüber künstlicher Intelligenz. | *Affektiv* | Negativ (reverse-coded) |
| **3** | Ich möchte Technologien nutzen, die auf künstlicher Intelligenz basieren. | *Behavioral* | Positiv |
| **4** | Künstliche Intelligenz hat mehr Nachteile als Vorteile. | *Kognitiv* | Negativ (reverse-coded) |
| **5** | Ich freue mich auf zukünftige Entwicklungen im Bereich künstliche Intelligenz. | *Affektiv* | Positiv |
| **6** | Künstliche Intelligenz bietet Lösungen für viele globale Probleme. | *Kognitiv* | Positiv |
| **7** | Ich bevorzuge Technologien, die keine künstliche Intelligenz beinhalten. | *Behavioral* | Negativ (reverse-coded) |
| **8** | Ich fürchte mich vor künstlicher Intelligenz. | *Affektiv* | Negativ (reverse-coded) |
| **9** | Ich würde mich eher für eine Technologie mit künstlicher Intelligenz entscheiden als für eine ohne. | *Behavioral* | Positiv |
| **10** | Künstliche Intelligenz verursacht eher Probleme, anstatt sie zu lösen. | *Kognitiv* | Negativ (reverse-coded) |
| **11** | Wenn ich an künstliche Intelligenz denke, habe ich hauptsächlich positive Gefühle. | *Affektiv* | Positiv |
| **12** | Ich möchte mit Technologien, die auf künstlicher Intelligenz beruhen, lieber nichts zu tun haben. | *Behavioral* | Negativ (reverse-coded) |
